# Synergistic antitumor effect of AAV-mediated TRAIL expression combined with cisplatin on head and neck squamous cell carcinoma

**DOI:** 10.1186/1471-2407-11-54

**Published:** 2011-02-03

**Authors:** Minghong Jiang, Zheng Liu, Yang Xiang, Hong Ma, Shilian Liu, Yanxin Liu, Dexian Zheng

**Affiliations:** 1 National Key Laboratory of Medical Molecular Biology, Institute of Basic Medical Sciences, Chinese Academy of Medical Sciences & Peking Union Medical College, Beijing 100005, China

## Abstract

**Background:**

Adeno-associated virus-2 (AAV-2)-mediated gene therapy is quite suitable for local or regional application in head and neck cancer squamous cell carcinoma (HNSCC). However, its low transduction efficiency has limited its further development as a therapeutic agent. DNA damaging agents have been shown to enhance AAV-mediated transgene expression. Cisplatin, one of the most effective chemotherapeutic agents, has been recognized to cause cancer cell death by apoptosis with a severe toxicity. This study aims to evaluate the role of cisplatin in AAV-mediated tumor necrosis factor-related apoptosis-inducing ligand (TRAIL) expression and the effect on HNSCC both in vitro and in vivo.

**Methods:**

Five human HNSCC cell lines were treated with recombinant soluble TRAIL (rsTRAIL) and infected with AAV/TRAIL to estimate the sensitivity of the cancer cells to TRAIL-induced cytotoxicity. KB cells were infected with AAV/EGFP with or without cisplatin pretreatment to evaluate the effect of cisplatin on AAV-mediated gene expression. TRAIL expression was detected by ELISA and Western blot. Cytotoxicity was measured by MTT assay and Western blot analysis for caspase-3 and -8 activations. Following the in vitro experiments, TRAIL expression and its tumoricidal activity were analyzed in nude mice with subcutaneous xenografts of HNSCC.

**Results:**

HNSCC cell lines showed different sensitivities to rsTRAIL, and KB cells possessed both highest transduction efficacy of AAV and sensitivity to TRAIL among five cell lines. Preincubation of KB cells with subtherapeutic dosage of cisplatin significantly augmented AAV-mediated transgene expression in a heparin sulfate proteoglycan (HSPG)-dependent manner. Furthermore, cisplatin enhanced the killing efficacy of AAV/TRAIL by 3-fold on KB cell line. The AAV mediated TRAIL expression was observed in the xenografted tumors and significantly enhanced by cisplatin. AAV/TRAIL suppressed the tumors growth and cisplatin augmented the tumoricidal activity by two-fold. Furthermore, Combination treatment reduced cisplatin-caused body weight loss in nude mice.

**Conclusion:**

The combination of AAV-mediated TRAIL gene expression and cisplatin had synergistic therapeutic effects on head and neck cancers and reduced the potential toxicity of cisplatin. These findings suggest that the combination of AAV/TRAIL and cisplatin may be a promising strategy for HNSCC therapy.

## Background

Head and neck cancer actually includes many different malignancies. The most common type of cancer in the head and neck is squamous cell carcinoma (HNSCC), originating from the mucosal epithelium of the nose, mouth and throat [1]. Local control of HNSCC is essential, and administering anticancer drugs directly into various lesion sites by injection is comparatively easy [2]. Therefore, adaptation of traditional chemotherapeutics to local and regional administration techniques in treating head and neck cancers is actively pursued to provide higher local concentrations of otherwise systemically toxic drugs. Cisplatin is one of most successful chemotherapeutic drugs of choice for head and neck cancers; however, it produces major toxicities to normal cells and organs at the concentrations necessary for effective treatment of malignancies [3,4]. A combination of cisplatin with other therapeutic method, such as gene therapy, has become attractive treatment project [5,6].

Gene therapy is considered to have an enormous potential benefit and involves various delivery vehicles that can transfer therapeutic genes to tumor cells. Adeno-associated virus (AAV) is a small virus which has attracted considerable interest from gene therapy researchers due to a number of features, such as lack of pathogenicity, infection non-dividing cells and stably integration into the host cell genome at a specific site in the human chromosome. AAV also presents very low immunogenicity, seemingly restricted to generation of neutralizing antibodies. These features make AAV a very attractive candidate for creating viral vectors for gene therapy [7,8]. To date, AAV vectors have been used for many clinical trials for treatment of some kinds of tumors [9-11].

AAV vectors have a broad host range and can transduce head and neck cancer cells [12]. However, an obstacle to these applications is low transgene expression efficiency, mainly due to a limited second strand synthesis [13,14]. Previous studies reported that DNA-damaging agents, such as UV light, gamma irradiation, cis-platinum, and tritiated thymidine can significantly increase the efficiency of AAV transduction in various cells, including nondividing cells [15], airway cells [16], neuronal cells [17] and cancer cells [12,18,19]. Cisplatin is an alkylating agent that targets DNA and results in bulky adducts as well as intra- and inter-strand crosslink [20,21]. Based on these promising characters, an alternative therapeutic modality, combination therapy of AAV-mediated gene expression and cisplatin might be a feasible candidate for HNSCC therapy.

Now being investigated for the treatment of HNSCC includes many gene transfer strategies that involve introduction of genes that directly kill tumor cells, restore of a defective tumor-suppressor gene, or trigger tumor apoptosis. Tumor necrosis factor (TNF)-related apoptosis-inducing ligand (TRAIL) is a potent apoptosis inducer that limits tumor growth without damaging normal cells and tissues in vivo and in vitro [22]. Mohr *et al*. demonstrated that long-term expression of TRAIL mediated by AAV led to a marked suppression of colorectal tumors in mouse with a single intratumoral injection of AAV/TRAIL vector [23]. We previously showed that AAV-mediated TRAIL gene therapy significantly suppressed the growth of human tumor cells transplanted in the liver [24,25] and lung [26] of mouse models.

In the present study, we investigated the role of cisplatin in AAV-mediated TRAIL gene therapy and its cytotoxic effect on HNSCC. We demonstrated that application of cisplatin significantly facilitated AAV/TRAIL transfer and TRAIL-induced apoptosis in HNSCC KB cell line in vitro and this combination significantly suppressed tumor growth in nude mice inoculated with KB subcutaneously. Furthermore, AAV/TRAIL was found to protect tumor-bearing mice from cisplatin-caused body weight loss. These findings suggest that combination of cisplatin and AAV/TRAIL may have synergistic therapeutic effects on the treatment of HNSCC patients.

## Methods

### Cell culture and reagents

Five human HNSCC cell lines were used to estimate the sensitivity to TRAIL. Human adenoid cystic carcinoma cell line Acc-2, tongue squamous cell carcinoma TCA and laryngeal carcinoma cell line Hep-2 were purchased from the Cell Culture Centre of Institute of Basic Medical Sciences, Chinese Academy of Medical Sciences (Beijing) and cultured in RPMI-1640 medium (Gibco BRL, Grand Island, NY) with 10% fetal bovine serum (FBS) (Hyclone, South American origin). Human oral squamous carcinoma cell line KB and nasopharyngeal carcinoma cell line CNE were purchased from China Center for Type Culture Collection, Wuhan University, and cultured in modified Eagle's medium with 2 mM L-glutamine and 0.1 mM non-essential amino-acids (MEM-NEAA) (Cell Culture Centre of Institute of Basic Medical Sciences, Chinese Academy of Medical Sciences, Beijing), 1.0 mM sodium pyruvate, 100 U/mL penicillin, 100 μg/mL streptomycin sulfate and 10% FBS. All cell cultures were maintained at 37°C in a humidified atmosphere of 5% CO_2_. Immunophenotyping heparin (10,000 U/mL; Changzhou Qianhong Bio-pharma Co., Ltd., Changzhou) was used for blocking infectious AAV experiments.

### Construction of AAV/TRAIL and AAV/EGFP vectors

AAV/TRAIL and AAV/EGFP were constructed as previously described [24] with a little of modification. Briefly, TRAIL_95-281 _cDNA with a woodchuck hepatitis B virus post-transcriptional regulatory element (WPRE) was amplified by PCR and then inserted into an AAV expression vector under control of CAG (cytomegalovirus enhancer plus chicken β-actin) promoter. Recombinant AAV vector encoding enhanced green fluorescent protein (AAV/EGFP) was constructed as control. Recombinant AAV viral particles were generated and purified as described [24].

### Cell infection with AAV/TRAIL and treatment with cisplatin

Adherent KB cells were removed from culture flasks and seeded in the cell culture plates at a density of 1 × 10^4^/mL. After an overnight culture, the medium was removed and replaced with the complete medium with the absence or presence of cisplatin (200 ng/mL) (Qilu pharmaceutical Co., Ltd, Shandong). Two hours later, the cells were infected with AAV/TRAIL and AAV/EGFP viruses at a multiplicity of infection (MOI) of 1 × 10^5 ^infectious particles per cell in FBS free and antibiotics free medium in the absence or presence of cisplatin. Four hours later, the medium was replaced with the complete medium in the absence or presence of cisplatin, and the cells went on being incubated for 72 h.

To assess the gene transduction in the presence of soluble heparin sulfate, a competitive inhibitor of the interaction between AAV and its primary attachment receptor, the cell surface molecule heparin sulfate proteoglycan (HSPG) was used to determine whether the robust gene transfer seen in KB cells was in fact due to their residual HSPG expression or other cell surface molecules. AAV/EGFP was incubated with 500 IU soluble heparin sulfate for 2 h at 37°C, and then the mixture was subjected to KB cells as control.

### Cell viability assay

Cell viability was quantified by a short-term microculture tetrazolium (MTT) assay. In a 96-well microplate, 1 × 10^4 ^cells per well were exposed to the treatments with recombinant soluble TRAIL (rsTRAIL) (Xingpeng Biotech Co., Shenzhen) for 24 h, cisplatin alone, AAV/TRAIL alone or the combination of both for 72 h, respectively. The media were replaced with 90 μL of free-serum medium and 10 μL of MTT solution (5 mg/mL in sterile phosphate-buffered saline). After 4-hour incubation at 37°C, the MTT solution in the wells was replaced with 100 μL dimethyl sulfoxide (DMSO). The absorption at 570 nm (OD570) was measured on a spectrophotometer. The cell viability was converted and expressed as the percentage of the control. Results were expressed as the mean OD for selected paradigms performed in duplicate (n = 3). The cells without any treatment were used as negative control.

### ELISA

The TRAIL concentrations in culture medium of KB cells with various treatments were measured by using a Quantikine human TRAIL/TNFSF10 immunoassay kit (R&D Systems; Minneapolis, MN) according to the manufacturer's instructions. The absorbance at 490 nm (OD490) was measured on a microplate reader.

### Western blot assay

To detect caspase activation in TRAIL-induced apoptosis, KB cells was pretreated with pan-caspase inhibitor Z-VAD-fmk followed by treatment with cisplatin and/or AAV/TRAIL. The cells were lysed for 30 min at 4°C in PBS with 1% NP-40 and protease inhibitor cocktail tablet (Roche, Mannheim) followed by high-speed centrifugation. Protein concentration was assayed by using bicinchoninic acid (Pierce, Rockford, IL). Total proteins were separated on a 10% SDS-PAGE. Immunodetection of TRAIL, caspase 8, caspase 3 and GAPDH was carried out by using rabbit anti-TRAIL antibody (Santa Cruz Biotechnology, Santa Cruz, CA), mouse anti-caspase 8 and rabbit anti-caspase 3 (Cell Signaling Technology, Danvers, MA) at 4°C overnight. The proteins in the gel were transferred onto a polyvinylidene difluoride (PVDF) membrane, and the membrane was incubated at 4°C for 16 h with the primary antibody. After three washes with TBST, the membrane was incubated with biotinylated goat anti-mouse IgG or goat anti-rabbit IgG for 1 h at room temperature. After thoroughly washing with TBST, the specific protein was visualized with enhanced luminescence reagents (Hyperfilm ECL, Amersham Biosciences, Buckinghamshire) followed by exposure to X-ray film for 3 min. Equal amount of protein loading was controlled by GAPDH in the sample and visualized with mouse anti-GAPDH mAb (Zhongshan Goldenbridge Biotechnology Co. Ltd, Beijing).

### Immunochemistry assay

Cryosections (10 μm thickness) prepared from tumors were incubated with the specific primary anti-TRAIL antibody (Santa Cruz Biotechnology, Santa Cruz, CA) overnight, washed, subsequently incubated for 30 min with appropriate HRP-conjugated goat anti-rabbit IgG secondary antibody (Santa Cruz Biotechnology, Santa Cruz, CA) in blocking buffer and DAB (Vector Laboratories, Burlingame, CA) as a substrate for the visualization of antigen-antibody complex.

### KB metastatic models and assessment of tumor growth in vivo

Four to six week's old male BALB/c nude mice with average weight of about 18 g were provided by the Institute of Zoological Sciences, Chinese Academy of Medical Sciences, Beijing. KB cells were infected in vitro for four hours with AAV/TRAIL or AAV/null at 1 × 10^4 ^v.g./cell, and the AAV/TRAIL or AAV/null infected cells (1.5 × 10^6^) were inoculated into the right dorsal flanks of the mice (n = 6). The tumor volume was measured with calipers twice weekly for length and width, and then calculated by the formula of [(length × width^2^)/2]. TRAIL expression in tumor tissues was checked by ELISA.

To evaluate the anti-tumor effect of AAV/TRAIL in vivo, 1.5 × 10^6 ^KB cells were injected subcutaneously at right back of nude mice. When the tumor volumes reached approximately 60 mm^3 ^on the seventh day postinjection, the animals were divided into four groups (n = 6) and two groups of animals were given AAV/TRAIL (1 × 10^10 ^v.g./100 μL) intratumorally or cisplatin (6 mg/kg) intraperitoneally (i. p.), respectively. One group of the animals was treated with 6 mg/kg cisplatin plus 1 × 10^10 ^v.g of AAV/TRAIL simultaneously. The control group of the animals was injected with AAV/null intratumorally and PBS i.p. injected. The tumor volume was measured and calculated. The animals were euthanized on the 33^th ^day post-treatment, and the tumors were processed for histology analysis. Mouse sera were collected for kidney function analysis of blood urea nitrogen (BUN) and creatinine (Cr) using an AU2700 Automatic Biochemical Analyzer (Olympus Co., Ltd.). All animal experiments were approved by the Committee of Use and Care of Animals, Chinese Academy of Medical Sciences.

### Statistical analysis

Data are presented as mean ± SD. Statistical analysis was conducted using the software package Minitab (version 15.1.0.0; Minitab, State College, PA). Significance was assessed by Student's *t *test and one-way ANOVA followed by Bonferroni *t *test for the comparisons of multiple means, or by Kruskal-Wallis test and subsequent pair-wise comparisons. The *p *value was considered to be statistically significant when less than 0.05.

## Results

### Human head and neck cancer cell lines possess different sensitivities to rsTRAIL

**Figure 1:**
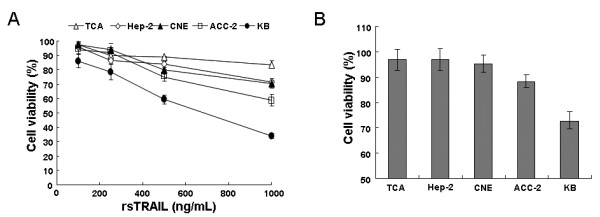
**Effects of rsTRAIL and AAV/TRAIL on cell viability in HNSCC cell lines**. (A) Treatment with rsTRAIL (50-1000 ng/mL) for 24 h; (B) transfected with AAV/TRAIL (MOI = 1 × 10^5 ^v.g.) for 72 h. Values are mean of three independent experiments with error bar representing standard deviation of the mean.

We first examined the sensitivity of HNSCC cell lines to rsTRAIL. As shown in Figure 1A, treatment with various concentrations of rsTRAIL for 24 h caused various viability reductions in five head and neck cell lines. Apoptosis in TCA, Hep-2 and CNE cells was about 17-30% at the concentration of 1.0 μg/mL rsTRAIL, suggesting these cells are resistant to rsTRAIL killing. Acc-2 was about 40% apoptosis under the treatment of rsTRAIL (1.0 μg/mL), showing a mild sensitivity to rsTRAIL. The viability of KB cells were 40% and 70%, respectively, in the presence of rsTRAIL of 0.5 and 1.0 μg/mL, indicating that KB is the highest susceptible to rsTRAIL cytotoxicity among the five HNSCC cell lines.

To further assess the sensitivity of cell lines to AAV-mediated TRAIL cytotoxicity the five cell lines were infected by AAV/TRAIL particles for 72 hours, respectively. As shown in Figure 1B, the viability of TCA, Hep-2 and CNE cells were about 90%, while KB cells 70%, suggesting that KB cells are both relatively high transduction efficacy of AAV and high sensitivity to AAV-mediated TRAIL cytotoxicity. Accordingly, we used KB as human HNSCC model to determine the efficacy of combination of AAV/TRAIL and cisplatin.

### Cisplatin enhances AAV-mediated EGFP expression in KB cells in a HSPG-dependent manner

**Figure 2:**
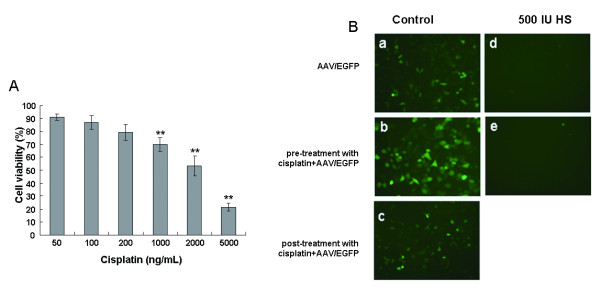
**Cisplatin exhibits dose-dependent cytotoxicity and augments AAV/EGFP transduction in KB cell line**. (A) Treatment with cisplatin (50-5000 ng/mL) for 72 h. (B) Fluorescence microscopy of KB cells without (a) or with treatment of cisplatin for 2 h before (b) and after (c) AAV/EGFP infection. For the competition experiments, KB cells were infected with the virus and incubated for 2 h at 37°C in the presence of 500 IU heparin sulfate (d and e). Values are mean of three independent experiments with error bar representing standard deviation of the mean.

To evaluate the effect of cisplatin on AAV-mediated gene expression, KB cells infected with AAV/EGFP particles were treated with cisplatin. The inhibitory rate of cisplatin alone was first tested to determine a sub-toxic dosage that induces minimal cell death (less than 25%). As indicated in Figure 2A, cisplatin induced cell death in a dose-dependent manner during 72 hour's treatment in KB cells. When the cells were treated with cisplatin of 200 ng/mL for 72 hours, the viability of KB dropped to 80% of untreated cells, indicating that 200 ng/mL of cisplatin is the sub-toxic dosage and then adapted for the combination with AAV/TRAIL.

Then, AAV transduction in response to cisplatin treatment was evaluated using the viral AAV/EGFP. KB cells were pre-incubated with cisplatin for 2 h followed by infection with AAV/EGFP. As shown in Figure 2B(a-c), EGFP expression was markedly increased compared to either without cisplatin or with cisplatin treatment post AAV/EGFP transduction, suggesting that cisplatin could enhance infection of AAV in KB cells. To determine whether cisplatin was capable of inducing AAV transduction in various HNSCC cell lines, four cell lines except KB were infected with AAV/EGFP and/or cisplatin. The results showed that these cisplatin-treated HNSCC cells all became susceptible to AAV/EGFP infection in comparison with untreated control (data not shown).

To explore whether cisplatin could enhance AAV-binding to its primary attachment receptor HSPG, 1 × 10^5 ^v.g. of AAV/EGFP were mixed with 500 IU heparin, an endogenesis inhibitor of AAV infection, and incubated with KB cells for 48 h. As shown in Figure 2Bd and 2Be, transduction of KB cells was completely blocked by heparin, no matter in the presence or absence of cisplatin, suggesting that cisplatin-induced AAV-mediated EGFP expression in KB cells was in a HSPG-dependent manner and not involved in additional cell surface molecules mediating attachment and entry of AAV.

### Cisplatin pre-treatment increases AAV/TRAIL-induced apoptosis in KB cells

**Figure 3:**
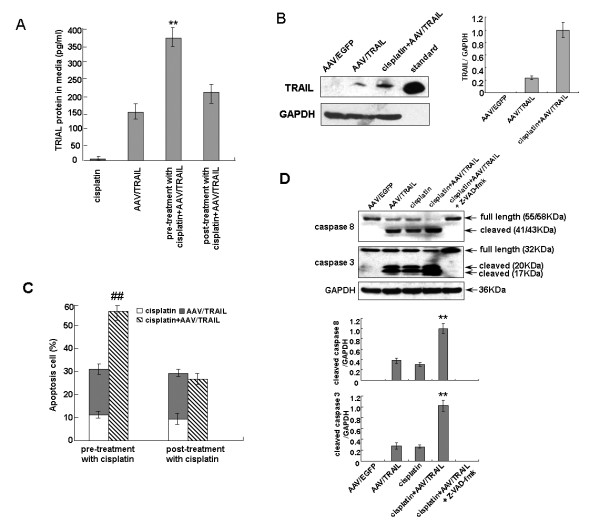
**Cisplatin enhances AAV-mediated TRAIL expression and apoptosis in KB cells**. KB cells pre-treated or post-treated with cisplatin were transducted with AAV/TRAIL for 72 h, the cells were harvested and TRAIL expression was analyzed by (A) ELISA assay and (B) Western blot assay. Purified rsTRAIL was used as a standard. The cells pre-treated with cisplatin (200 ng/mL for 2 h) and apoptosis was detected by MTT assay (C) and (D) Western blot analysis for the activation of caspase-8 and -3 in KB cells. Densitometric analysis showing change in optical density of TRAIL, cleaved caspase-3 and -8 bands. GAPDH expression was used as adjustment. Values are mean of three independent experiments with error bar representing standard deviation of the mean. **P < 0.01 compared to the values obtained with the equivalent dose of AAV/TRAIL alone. ##*P *< 0.01 compared to the sum effect of AAV/TRAIL and cisplatin.

To evaluate the potential effect of the combination of AAV/TRAIL and cisplatin on HNSCC cells, TRAIL expression and its cytotoxicity were determined. As shown in Figure 3A, TRAIL expression in KB cells treated with cisplatin (200 ng/mL) for 2 h followed by AAV/TRAIL infection was increased 2.3-fold over AAV/TRAIL alone, and 1.75-fold over AAV/TRAIL transduction first and followed by cisplatin. Effects of treatment with the combination on TRAIL expression in KB cells were assessed by Western blot analysis. The combination of AAV/TRAIL and cisplatin significantly increased TRAIL expression compared to either treatment (Figure 3B). Pre-treatment with cisplatin and followed by AAV/TRAIL in KB cells caused 57.6% inhibition of the cell viability, which was much higher than either cisplatin (11%) or AAV/TRAIL (20%) only, or AAV/TRAIL transduction first and followed by cisplatin (28%) (Figure 3C), indicating that there is a synergetic effect of the combination treatment on the viability of KB cells. To confirm the cell death property, the activated forms of caspase-3 and caspase-8 were measured by Western blot analysis. As shown in Figure 3D, the intensities of cleaved caspase-3 and -8 bands were almost similar in KB cells treated with AAV/TRAIL or cisplatin alone. Treatment with cisplatin and AAV/TRAIL resulted in a significant increase in the activation of caspase-3 and -8 over either of them alone. Additionally, pre-incubation of KB with the caspase inhibitor z-VAD-fmk completely prevented cleavage of caspase-3 and -8. These results suggest that cisplatin pre-treatment increases AAV/TRAIL-induced apoptosis profoundly in KB cells.

### Combination of AAV/TRAIL and cisplatin suppresses the growth of HNSCC xenografts in nude mice

**Figure 4:**
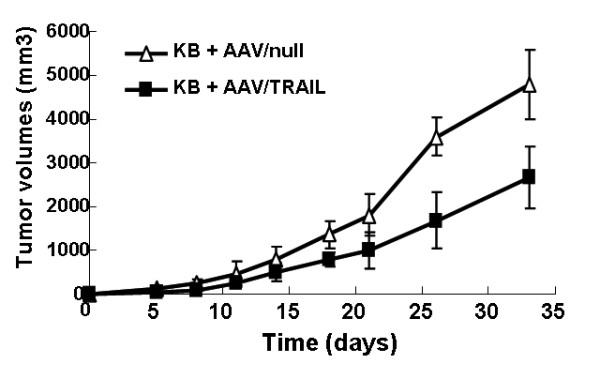
**Infection of AAV/TRAIL suppresses tumor formation and growth in BALB/c nude mice**. KB cells were infected with AAV/TRAIL or AAV/null for 4 h in vitro, and then injected into nude mice subcutaneously. The tumor formation was observed for 33 days. Values are mean with error bar representing standard deviation of the mean (n = 6).

We further investigated the effect of the combination of AAV/TRAIL and cisplatin on tumor formation and growth in nude mice transplanted with KB cells. KB cells infected with AAV/TRAIL or AAV/null were subcutaneously inoculated into the right dorsal flanks of nude mice and tumor formation was monitored twice weekly during a period of 33 day's experiment. Notably, the mean volume of the tumors generated from the KB cells infected with AAV/TRAIL was about 45% of that generated from the cells infected with AAV/null (Figure 4). ELISA analysis revealed that TRAIL expression in the tumors infected with AAV/TRAIL on the 21^st ^day was about 70 ng/mg tissue, but not in the tumors infected with AAV/null, suggesting that AAV-mediated TRAIL expression effectively suppresses tumor formation in the animal model.

**Figure 5:**
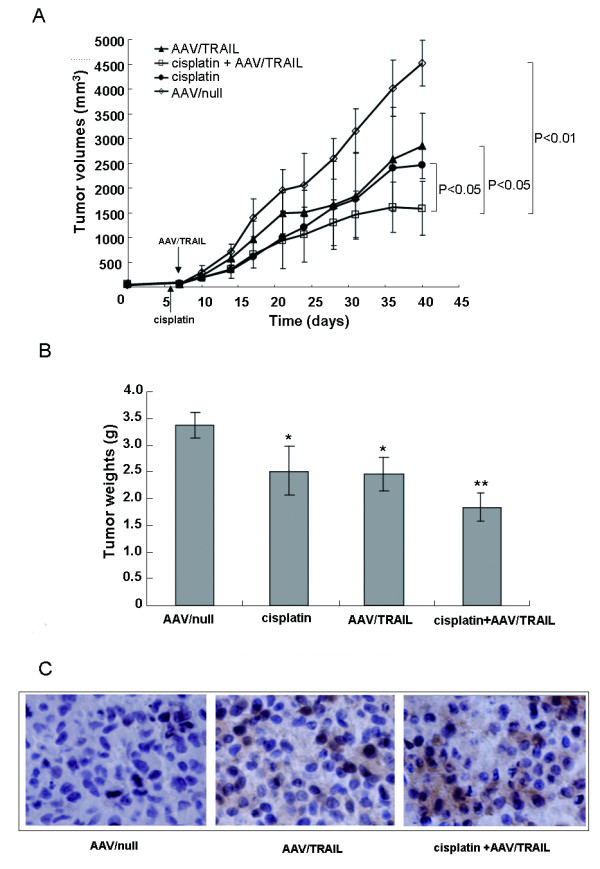
**Synergistic antitumor effect of the combination of AAV/TRAIL and cisplatin in vivo**. (A) KB cells were inoculated into the right thighs of BALB/c nude mice. When the tumor size was reached to 60 mm^3^, the mice were received an intratumoral injection of AAV/TRAIL, intraperitoneal injection of cisplatin and the combination treatment. The tumor volume was measured and calculated. The animals were euthanized on the day 33 post-treatment. The tumor weights were determined (B) and processed for immunohistochemistry analysis (× 200) (C). Values are mean with error bar representing standard deviation of the mean (n = 6). *P < 0.05, **P < 0.01 compared to AAV/null.

We next attempted to find out whether cisplatin pretreatment followed by AAV/TRAIL infection was effectively blocked the tumor growth in an established HNSCC mouse model. KB cells were subcutaneously injected into the right thighs of nude mice. When reaching an average tumor volume of about 60 mm^3^, the mice were randomly divided into 4 groups (n = 6) administrated with PBS plus AAV/null as control, AAV/TRAIL, cisplatin, and the combination of AAV/TRAIL plus cisplatin, respectively. On the 14^th ^day of post-treatment, the tumors in the mice treated with the combination of AAV/TRAIL and cisplatin began to grow slower than the other three groups. On the 33^rd ^day of post-treatment, the tumor size in the combination group was inhibited by 65%, to a much greater extent than that of mice treated with the intratumor injection of AAV/TRAIL only (36%) or single intraperitoneal administration with cisplatin (21%) (Figure 5A). The tumor weights in the combination group were significantly lower than that treated with either of them (*P *< 0.05) and AAV/null group (*P *< 0.01) (Figure 5B). Histological analysis showed a substantial number of TRAIL positive cells in the tumor tissue infected with AAV/TRAIL alone and combination of AAV/TRAIL plus cisplatin, but few in the tumor tissue infected with AAV/null (Figure 5C). These data indicate that combination of AAV/TRAIL and cisplatin suppresses human tumor formation and growth significantly in nude mice.

### AAV/TRAIL reduces cisplatin-caused body weight loss in nude mice

**Figure 6:**
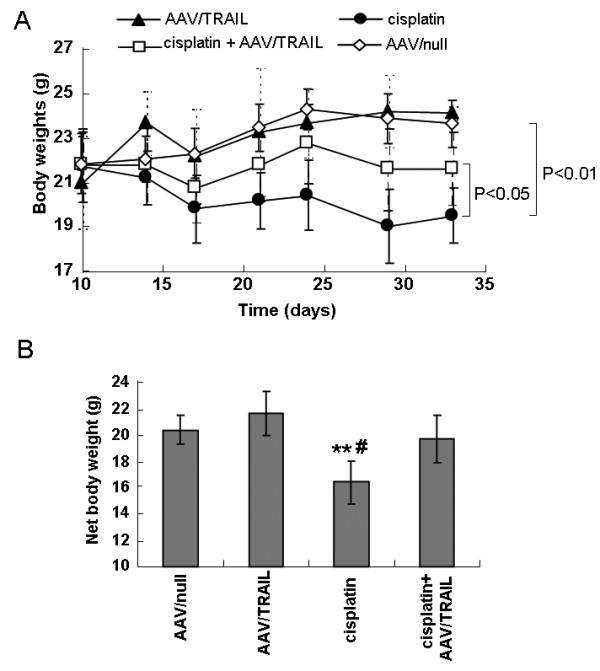
**The changes of body weight (A) and net body weight (the tumor weight subtracted from the total body weight) (B) of the mice in the respective treatment groups**. Values are mean with error bar representing standard deviation of the mean (n = 6). **P < 0.01 compared to AAV/null, #*P *< 0.05 compared to the combination group.

Interestingly, we observed that the animals received cisplatin 6 mg/kg, i.p. showed a decrease in their body weight by 2.2 ± 0.8 g compared to AAV/null and AAV/TRAIL treated animals, which gained 1.8 ± 0.5 g body weight (*P *< 0.01) during the period of 33 day's experiment, and the animal body weight in the combination group was almost no change (Figure 6A). To eliminate the effect of tumor weight on the body weight, net body weight of the mice, namely the tumor weight subtracted from the whole body weight, was calculated. As shown in Figure 6B, the net body weight in the cisplatin group was the lowest among the three groups and there was statistically significant difference compared with AAV/null group (*P *< 0.01) and the combination group (*P *< 0.05).

## Discussion

Goals of cancer treatment generally consist of removal of cancer load, maintenance of quality of life, and prevention of subsequent primary tumors. Despite improved chemotherapeutic strategies, resistance and toxic side effects of many tumors such as head and neck cancers to current treatment protocols remain major concerns in tumor therapy. Therefore, more effective therapeutic modalities, being selective for tumor cells without toxicity for normal cells, are preferred. Head and neck cancers are accessible to intratumoral injection therapy and good candidates for trials of gene therapy. The recombinant vectors based on AAV, a non-pathogenic virus with a single-stranded DNA genome, have a broad host range and can transduce head and neck cancer cells, so that they are currently being used in a number of clinical trials and may become an important component for the therapy of tumor patients [9-11]. However, a major challenge of AAV-mediated gene therapy is to improve its transduction and expression efficiency in targeted tissues. In the present study, we report that chemotherapeutic drug cisplatin enhanced AAV vector-mediated TRAIL expression and cytocidal effect on HNSCC cells. This finding was confirmed in animal model with xenografts of HNSCC that cisplatin enhanced AAV-mediated TRAIL expression and augmented the antitumor activity of AAV/TRAIL, suggesting that combination of AAV/TRAIL and cisplatin is significantly effective in the treatment of HNSCC and may lead to reduction of the cisplatin toxicity. It is reasonable to expect the use of AAV/TRAIL for local or regional delivery to bulky cancers in conjunction with systemic cisplatin-based chemotherapy for optimal and synergistic antitumor effect.

As a chemotherapeutic agent, cisplatin is preciously reported to significantly enhance AAV transduction in some kinds of cells. However, whether cisplatin is able to induce AAV transduction in squamous cell carcinoma cells has never been tested. In the present study, we evaluated the AAV transduction in response to cisplatin treatment using AAV/EGFP viral particles. Pretreating KB cells with subtherapeutic dosage of cisplatin before AAV infection resulted in a significant enhancement of transgene expression of both AAV/EGFP and AAV/TRAIL. However, the underlying molecular mechanism of this phenomenon has not been clearly elucidated. Membrane-associated HSPG, a principal attachment receptor for AAV mediating both attachment and infection of target cells, has been reported [27]. It is not known whether the increased AAV transduction when combined with cisplatin would be mediated by similar receptor. We conducted further experiments to address this possibility. Inhibition of AAV attachment and infection was shown to be achieved in competition experiment using heparin sulfate, a molecule chemically very similar to HSPGs thus functioning as a soluble receptor analog. Transgene expression was significantly blocked by pre-incubation of the cells with heparin sulfate in KB cells in the presence or absence of cisplatin, suggesting that cisplatin-induced AAV permissiveness in KB cells is a normal HSPG-dependent cell entry pathway. For the potential mechanism of the synergistic effect on AAV transduction, previous studies reported that cells exposed to DNA-damaging agents might provide the sites for AAV integration or lead to induction of repair enzymes and factors modulating cell cycle progression [15]. The machinery of cellular DNA repair synthesis may play an important role in converting the single-stranded AAV vector genome to a double-stranded form, which is activated by cisplatin [13,28]. Platinum-based cisplatin is the first member of a class of anti-cancer drugs which react *in vivo*, binding to and causing cross-linking of DNA which ultimately triggers apoptosis. Thus, it is possible that the cisplatin-induced AAV permissiveness is contributed in part by overcoming barriers for the second strand DNA synthesis. Increased transduction appears to reflect an increase in the number of input vector genomes converted to a form that allows gene expression [15].

As predicted, combination of AAV-mediated TRAIL expression and cisplatin enhanced the cytotoxicity and apoptosis efficacy in KB cancer cells. However, it was not clear whether there were other factors except increased TRAIL protein likely responsible for the enhancement of cisplatin-induced apoptosis. In previous studies, it was shown that the cytotoxicity efficacy of TRAIL was enhanced by cisplatin [29-34]. Furthermore, AAV infection alone was not sufficient to trigger apoptosis, but that in combination with cisplatin, it could enhance cell death in HeLa and A549 cells [35]. In the present study, we failed to demonstrate a synergistic cytotoxic effect neither the post-treatment of cisplatin after AAV/TRAIL infection (Figure 3C) nor the combination of rsTRAIL and various dosages of cisplatin in KB cells (Additional file 1 Figure S1). So the main mechanism underlying of the cisplatin-mediated enhancement of AAV cytotoxic efficacy might be only involved in cisplatin-induced AAV-mediated TRAIL expression in KB cells.

The synergistic tumoricidal activity of AAV-mediated TRAIL and cisplatin in KB cells prompted us to evaluate the therapeutic efficacy in animal models. It was observed that TRAIL protein expression at high level effectively suppressed both tumor formation and growth in nude mice transplanted with KB cells pre-infected with AAV/TRAIL in vitro. Moreover, in the other tumor model of intratumor delivery of AAV/TRAIL, our data demonstrated that the combination of AAV/TRAIL gene therapy and chemotherapeutic agent cisplatin suppressed tumor growth more efficiently than either alone, suggesting that the cytotoxicity mediated by AAV/TRAIL can be strengthened by cisplatin. However, we failed to detect TRAIL protein in tumor tissues by immunoblotting or ELISA using anti-TRAIL antibody in this model. Nevertheless, we observed significant inhibition of tumor growth (Figure 5A and 5B), suggesting that the biologically active TRAIL was expressed even at very low level in tumors. Yoo J et al. [36] also could not detect secreted TRAIL (sTRAIL) protein in mice sera after intravenous delivery of rAAV-sTRAIL via the tail vein, but they did observe a number of apoptotic tumor cells and significant inhibition of tumor growth, suggesting that TRAIL could be secreted into the body circulation and inhibit tumor formation at a distant site even at undetectable expression level. In our study, the secreted form of TRAIL was perhaps distributed inhomogeneously in relative big tumor tissues (about 2.5 grams) so that it was difficult for us to measure TRAIL protein in a little random cut tissues (100 mg or so). However, we successfully detected TRAIL expression in tumor tissues by immunochemistry assay and scanning of the whole tumor sections. In addition, it was interestingly to note that in the experiments using rsTRAIL a minimum of 100-200 ng/mL was needed to evaluate the apoptosis effects (Figure 1A and 2A), whereas AAV-mediated TRAIL expression yielding only 150 pg/mL reached similar cytotoxicity to HNSCC cells (Figure 3A). This difference might be caused by the different origins of TRAIL proteins, the former produced in bacteria (rsTRAIL) and the latter from AAV-mediated gene expression in eukaryotic cells where TRAIL could have its biological activity at very low concentration.

Despite cisplatin as one of the most widely used and most potent chemotherapy drugs, its side effects in normal tissues and organs limits its use and efficacy in cancer therapy. In the present study, intraperitoneal administration of cisplatin resulted in significant reduction of body weight. AAV/TRAIL was found to protect tumor-bearing mice from cisplatin-caused body weight loss. To further assess AAV/TRAIL protection from cisplatin toxicity, we administrated AAV/TRAIL systemically in normal nude mice without tumors. As a result, the body weights in the combination of AAV/TRAIL plus cisplatin group were significantly higher than the mice treated with single cisplatin (Figure 6), suggesting that the soluble TRAIL secreted into body circulation might attenuate cisplatin-caused systemic toxicity. We also detected the nephrotoxicity of cisplatin in nude mice assessed by blood urea BUN and Cr levels. While BUN and Cr levels were no significant changes among the experimental animals on the 33^rd ^day after cisplatin treatment (data not shown). The reason might be that low dose of cisplatin causes low nephrotoxicity and early recovery from the impaired renal function. Whether AAV/TRAIL protects against cisplatin-caused nephrotoxicity remains to be further clarified.

## Conclusions

In conclusion, we provided evidences in this study that cisplatin pretreatment can enhance transduction of AAV in HNSCC cells, up-regulate TRAIL expression mediated by AAV, and inhibit tumor cell growth in vitro. The combined administration of cisplatin and AAV/TRAIL significantly suppressed both tumor formation and growth of HNSCC xenografts in nude mice, protected against cisplatin-caused toxic symptoms. These findings indicate the potential of this combination of AAV/TRAIL and cisplatin as a novel therapeutic strategy for HNSCC. It seems likely, however, that gene transfer will find its way into the multidisciplinary care of the head and neck cancer patients, where novel treatments are combined with conventional therapies to maximize tumor response.

## Abbreviations

TRAIL: tumor necrosis factor-related apoptosis-inducing ligand; HNSCC: head and neck squamous cell carcinomas; HSPG: heparin sulfate proteoglycan.

## Competing interests

The authors declare that they have no competing interests.

## Authors' contributions

MHJ performed the experiments and wrote the manuscript. ZL carried out animal experiments and contributed to the evaluation of treatment effects and is co-first author. YX and HM participated in evaluating the histopathology and immunochemistry of the specimens. SLL and YXL participated in analyzing the data. DXZ, as the corresponding author, designed the protocol and revised the draft of the manuscript. All authors read and approved the final manuscript.

## Pre-publication history

The pre-publication history for this paper can be accessed here:

http://www.biomedcentral.com/1471-2407/11/54/prepub

## Supplementary Material

Additional file 1 **Effect of combining rsTRAIL with cisplatin *in vitro***. KB cells were treated with either rsTRAIL (400 ng/mL) or cisplatin (100-1000 ng/mL) or combination treatment for 24 h. Apoptosis was determined by MTT assay. Values are mean of three independent experiments with error bar representing standard deviation of the mean. Click here for file
