# Supplementary material for: Synergistic antitumor effect of AAV-mediated TRAIL expression combined with cisplatin on head and neck squamous cell carcinoma
Source: BMC Cancer. 2011 Feb 3;11:54. doi: 10.1186/1471-2407-11-54 (PMC3044652; doi:10.1186/1471-2407-11-54)
Supplement: Additional file 1 — Effect of combining rsTRAIL with cisplatin in vitro. KB cells were treated with either rsTRAIL (400 ng/mL) or cisplatin (100-1000 ng/mL) or combination treatment for 24 h. Apoptosis was determined by MTT assay. Values are mean of three independent experiments with error bar representing standard deviation of the mean. [file 1471-2407-11-54-S1.DOC]

**Additional files**

Additional file 1, Figure S1

**Title:** Effect of combining rsTRAIL with cisplatin *in vitro*.

**Description:** KB cells were treated with either rsTRAIL (400 ng/mL) or cisplatin (100-1000 ng/mL) or combination treatment for 24 h. Apoptosis was determined by MTT assay. Values are mean of three independent experiments with error bar representing standard deviation of the mean.


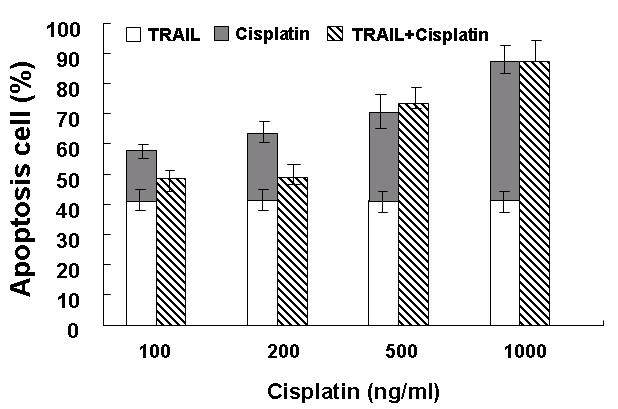


**Additional file 1, Figure S1**
